# Supplementary material for: What are Deaf sign language users’ experiences as patients in healthcare services? A scoping review
Source: PLOS Glob Public Health. 2025 Feb 26;5(2):e0003535. doi: 10.1371/journal.pgph.0003535 (PMC11864532; doi:10.1371/journal.pgph.0003535)
Supplement: S1 Table — (DOCX) [file pgph.0003535.s001.docx]

S1 Table. Full-text articles assessed for eligibility (n=91)

| **Authors (year)** | **Title** | **Included or Excluded** | **If excluded – reasons for excluded** | **Name of data extractor / date of data extraction** |
| --- | --- | --- | --- | --- |
| Adigun et al (2020) | 'They Forget I'm Deaf': Exploring the Experience and Perception of Deaf Pregnant Women Attending Antenatal Clinics/Care. | Included | N/A | Katherine Rogers / March 2024 |
| Adigun et al (2021) | Including the Excluded in Antenatal Care: A Systematic Review of Concerns for D/deaf Pregnant Women. <https://doi.org/10.3390/bs11050067> | Excluded | Wrong study design (e.g. a review with no primary data) | N/A |
| Albrecht et al (2015) | iSignIT - Communication App and Concept for the Deaf and Hard of Hearing. <https://doi.org/10.3233/978-1-61499-538-8-283> | Excluded | Wrong outcome | N/A |
| Allen et al (2002) | American Sign Language and end-of-life care: research in the deaf community. <https://doi.org/10.1023/a:1020508511133> | Excluded | Wrong outcome | N/A |
| Anderson et al (2017) | Deaf people's help-seeking following trauma: Experiences with and recommendations for the Massachusetts behavioral health care system. | Included | N/A | Katherine Rogers / March 2024 |
| Barnett (2002) | Cross-cultural communication with patients who use American Sign Language.  [URL link to the article](https://www.researchgate.net/profile/Steven-Barnett-4/publication/11333811_Cross-cultural_communication_with_patients_who_use_American_Sign_Language/links/5d1f669b458515c11c14fc78/Cross-cultural-communication-with-patients-who-use-American-Sign-Language.pdf) | Excluded | Wrong study design | N/A |
| Beaver & Carty (2021) | Viewing the healthcare system through a deaf lens. <https://doi.org/10.17061/phrp3152127> | Excluded | Wrong study design | N/A |
| Bentes et al (2021) | Deaf person's perception on health care in a midsize city: an descriptive-exploratory study. <https://doi.org/10.5935/1676-4285.20113210> | Excluded | Non relevant language | N/A |
| Berman et al (2013) | Breast cancer knowledge and practices among D/deaf women. | Included | N/A | Katherine Rogers / March 2024 |
| Berman et al (2017) | D/deaf Breast Cancer Survivors: Their Experiences and Knowledge. | Included | N/A | Katherine Rogers / March 2024 |
| Bown et al (2020) | Supporting patients who are deaf who use a signed language in general practice. <https://doi.org/10.3399/bjgp20X707285> | Excluded | Wrong study design | N/A |
| Cardoso et al (2006) | Perception of persons with severe or profound deafness about the communication process during health care. | Included | N/A | Katherine Rogers / March 2024 |
| Cerilli et al (2023) | "There is No Communication": A Qualitative Examination of Deaf Signers' Experiences With Advance Care Planning. | Included | N/A | Katherine Rogers / March 2024 |
| Chin et al (2013) | Deaf mothers and breastfeeding: do unique features of deaf culture and language support breastfeeding success? | Included | N/A | Katherine Rogers / March 2024 |
| Chong et al (2023) | The design of the Deaf in Touch Everywhere (DITE)TM mobile application with Deaf and interpreter communities in Malaysia. <https://doi.org/10.1177/20552076241228432> | Excluded | Wrong outcome | N/A |
| Coignard et al (2015) | Rethinking the Prescription's Comprehension: an Example of Care Centers for Deaf People. <https://doi.org/10.2515/therapie/2015034> | Excluded | Non relevant language | N/A |
| Costa et al (2018) | Welcome and listen to the silence: nursing care from the perspective of deaf woman during pregnancy, childbirth and postpartum. | Included | N/A | Katherine Rogers / March 2024 |
| Didero et al (2024) | Challenges of Being Deaf and Aging With HIV: Focus Group Findings From Palm Springs, California. <https://doi.org/10.1097/JNC.0000000000000317> | Excluded | Wrong population | N/A |
| Dock (2018) | HEALTH CARE ACCESS AMONG INDIVIDUALS WHO ARE DEAF. <https://link.gale.com/apps/doc/A694120970/HRCA?u=anon~89e154b1&sid=googleScholar&xid=401d0a01> | Excluded | Wrong study design | N/A |
| Ferguson et al (2003) | Communication needs of patients with altered hearing ability: Informing pharmacists' patient care services through focus groups. | Included | N/A | Katherine Rogers / March 2024 |
| Fernandez-Valderas et al (2017) | Experiences of deafblind people about health care. | Included | N/A | Katherine Rogers / March 2024 |
| Foltz & Shank (2020) | Deaf Sign-Language Using Patients' Experiences in Health Emergencies in Wales: Perspectives for Improving Interactions. | Included | N/A | Katherine Rogers / March 2024 |
| Gichane et al (2017) | "They must understand we are people": Pregnancy and maternity service use among signing Deaf women in Cape Town. | Included | N/A | Katherine Rogers / March 2024 |
| Gilchrist (2000) | Through the Looking Glass: The lived experiences of deaf people communicating with nurses, the meaning of health, and attitudes toward nurses. | Included | N/A | Katherine Rogers / March 2024 |
| Havercamp et al (2020) | Chronic Disease Self-Management Program in American Sign Language: Evaluation and Recommendations. <https://doi.org/10.1177/1524839918792030> | Excluded | Wrong outcome | N/A |
| Hall et al (2023) | Deaf patients′ preferred communication in clinical settings: implications for healthcare providers. <https://doi.org/10.1093/deafed/enad061> | Excluded | Wrong population | N/A |
| Helm et al (2023) | Maternal Health Experiences of Black Deaf and Hard of Hearing Women in the United States. <https://doi.org/10.1016/j.whi.2023.07.005> | Excluded | Wrong population | N/A |
| Hill et al (2020) | Assessing and Providing Culturally Competent Care in Radiation Oncology for Deaf Cancer Patients. <https://doi.org/10.1016/j.adro.2020.02.007> | Excluded | Wrong study design | N/A |
| Hocker et al (2012) | Are Deaf Patients in Germany Informed about their Legal Rights for a Sign Language Interpreter? Results of a Nation-Wide Cross-Sectional Study with Sign Language Videos. <https://doi.org/10.1055/s-0031-1299779> | Excluded | Non relevant language | N/A |
| Hubbard et al (2018) | Promoting Best Practice for Perinatal Care of Deaf Women. | Included | N/A | Katherine Rogers / March 2024 |
| Hulme et al (2021) | Exploring the lived experiences of British Sign Language (BSL) users who access NHS adult hearing aid clinics: an interpretative phenomenological analysis. <https://doi.org/10.1080/14992027.2021.1963857> | Excluded | Wrong outcome | N/A |
| Iezzoni et al (2004) | Communicating about health care: observations from persons who are deaf or hard of hearing. | Included | N/A | Katherine Rogers / March 2024 |
| Jacob et al (2021) | Design suggestions for an mHealth app to facilitate communication between pharmacists and the Deaf: perspective of the Deaf community (HEARD Project). | Included | N/A | Katherine Rogers / March 2024 |
| Jacobs et al (2021) | A Mixed Methods Study of Hysterectomy in a U.S. Sample of Deaf Women Who Use American Sign Language. | Included | N/A | Katherine Rogers / March 2024 |
| James et al (2022) | "They're Not Willing To Accommodate Deaf patients": Communication Experiences of Deaf American Sign Language Users in the Emergency Department. | Included | N/A | Katherine Rogers / March 2024 |
| James, Panko, et al (2023) | Healthcare communication access among deaf and hard-of-hearing people during pregnancy. | Included | N/A | Katherine Rogers / March 2024 |
| James, Sullivan, et al (2023) | Emergency department patient-centred care perspectives from deaf and hard-of-hearing patients. | Included | N/A | Katherine Rogers / March 2024 |
| Kehl et al (2010) | Can you hear me now? The experience of a deaf family member surrounding the death of loved ones. <https://doi.org/10.1177/0269216309348180> | Excluded | Wrong outcome | N/A |
| Kritzinger et al (2014) | "I just answer 'yes' to everything they say": Access to health care for deaf people in Worcester, South Africa and the politics of exclusion. <https://doi.org/10.1016/j.pec.2013.12.006> | Excluded | Wrong population | N/A |
| Kushalnagar et al (2019) | Video Remote Interpreting Technology in Health Care: Cross-Sectional Study of Deaf Patients' Experiences. | Included | N/A | Katherine Rogers / March 2024 |
| Kushalnagar et al (2020) | Prostate-specimen antigen (PSA) screening and shared decision making among deaf and hearing male patients. <https://doi.org/10.1007/s13187-018-1436-3> | Excluded | Wrong outcome | N/A |
| Kyle et al (2013) | Deaf Health: Analysis of the current health and access to health care of Deaf people in the UK. Part 1: Health Assessment and Quantitative Data. | Included | N/A | Katherine Rogers / March 2024 |
| Lee et al (2021) | Access and communication for deaf individuals in Australian primary care. | Included | N/A | Katherine Rogers / March 2024 |
| Lesch et al (2019) | Barriers to healthcare services and supports for signing deaf older adults. <https://doi.org/10.1037/rep0000252> | Excluded | Wrong study design | N/A |
| Luton et al (2022) | Deaf women's experiences of maternity and primary care: An integrative review. <https://doi.org/10.1016/j.midw.2021.103190> | Excluded | Wrong study design | N/A |
| MacKinney et al (1995) | Improvements in preventive care and communication for deaf patients: results of a novel primary health care program. | Included | N/A | Katherine Rogers / March 2024 |
| Mastrocinque et al (2017) | Deaf Victims’ Experiences With Intimate Partner Violence: The Need for Integration and Innovation. <https://doi.org/10.1177/0886260515602896> | Excluded | Wrong outcome | N/A |
| Mauffrey et al (2016) | Comment les patients Sourds perçoivent-ils leur prise en charge en médecine générale. Enquête qualitative [Qualitative survey of deaf patients concerning perception of their management by general practitioners]. <https://shs.cairn.info/revue-sante-publique-2016-2-page-213?lang=fr> | Excluded | Non relevant language | N/A |
| McKee et al (2011) | Perceptions of cardiovascular health in an underserved community of deaf adults using American Sign Language. <https://doi.org/10.1016/j.dhjo.2011.04.001> | Excluded | Wrong outcome | N/A |
| Middleton et al (2010) | Preferences for communication in clinic from deaf people: a cross-sectional study. <https://doi.org/10.1111/j.1365-2753.2009.01207.x> | Excluded | Wrong outcome | N/A |
| Miller et al (2019) | Deaf LGBTQ Patients' Disclosure of Sexual Orientation and Gender Identity to Health Care Providers. | Included | N/A | Katherine Rogers / March 2024 |
| Mussallem et al (2022) | Making virtual health care accessible to the deaf community: Findings from the telehealth survey. | Included | N/A | Katherine Rogers / March 2024 |
| Mustafa & Addar (2000) | Obstetric handling of a deaf patient.  [URL link to the article](https://citeseerx.ist.psu.edu/document?repid=rep1&type=pdf&doi=1ab5520e7bc7f4a291283cf6ad8a736dee8140d2#:~:text=The%20management%20of%20labor%20in,(due%20to%20childhood%20deafness).) | Excluded | Wrong study design | N/A |
| Myers et al (2022) | Access to Effective Communication Aids and Services among American Sign Language Users across North Carolina: Disparities and Strategies to Address Them. | Included | N/A | Katherine Rogers / March 2024 |
| Napier et al (2013) | English literacy as a barrier to health care information for deaf people who use Auslan. | Included | N/A | Katherine Rogers / March 2024 |
| Napier et al (2014) | Direct, Interpreter-Mediated or Translated? A Qualitative Study of Access to Preventive and Ongoing Healthcare Information for Australian Deaf People. | Included | N/A | Katherine Rogers / March 2024 |
| O'Hearn et al (2006) | Deaf women's experiences and satisfaction with prenatal care: a comparative study. | Included | N/A | Katherine Rogers / March 2024 |
| Oliveira et al (2015) | Accessibility of the deaf person to public health services. | Included | N/A | Katherine Rogers / March 2024 |
| Palese et al (2011) | One-dimensional scales for pain evaluation adopted in Italian nursing practice: Giving preference to deaf patients. <https://doi.org/10.1891/1061-3749.19.2.91> | Excluded | Wrong outcome | N/A |
| Panko et al (2021) | The Deaf Community's Experiences Navigating COVID-19 Pandemic Information. <https://doi.org/10.3928/24748307-20210503-01> | Excluded | Wrong outcome | N/A |
| Panko et al (2022) | Experiences of pregnancy and perinatal healthcare access of women who are deaf: a qualitative study. | Included | N/A | Katherine Rogers / March 2024 |
| Parise (1999) | Breaking Cultural Barriers to Health Care: The Voice of the Deaf. | Included | N/A | Katherine Rogers / March 2024 |
| Pereira & Fortes (2010) | Communication and information barriers to health assistance for deaf patients. | Included | N/A | Katherine Rogers / March 2024 |
| Pertz et al (2018) | Addressing Mental Health Needs for Deaf Patients Through an Integrated Health Care Model. | Included | N/A | Katherine Rogers / March 2024 |
| Pinilla et al (2019) | Primary non-communicable disease prevention and communication barriers of deaf sign language users: a qualitative study. | Included | N/A | Katherine Rogers / March 2024 |
| Rannefeld et al (2023) | Deaf and hard-of-hearing patients are unsatisfied with and avoid German health care: Results from an online survey in German Sign Language. <https://doi.org/10.1186/s12889-023-16924-w> | Excluded | Wrong population | N/A |
| Reeves et al (2005) | Communication and Communication Support in Primary Care: A Survey of Deaf Patients. | Included | N/A | Katherine Rogers / March 2024 |
| Rodriguez-Martin et al (2018) | Ethnographic analysis of communication and the deaf community's rights in the clinical context. | Included | N/A | Katherine Rogers / March 2024 |
| Royal College of Psychiatry (2017) | Guidance for commissioners of primary care mental health services for deaf people. <https://signhealth.org.uk/wp-content/uploads/2024/08/2017-Guidance-for-commissioners-of-primary-care-mental-health-services-for-deaf-people-Joint-Commissioning-Panel-for-Mental-Health.pdf> | Excluded | Wrong study design | N/A |
| Saunders & Oliver (2022) | Impact of Hearing Loss on Communication During Remote Health Care Encounters. <https://doi.org/10.1089/tmj.2021.0490> | Excluded | Wrong population | N/A |
| Schniedewind et al (2020) | Ask and ye shall not receive: Interpreter-related access barriers reported by Deaf users of American sign language. | Included | N/A | Katherine Rogers / March 2024 |
| Senayah et al (2019) | The accessibility of health services to young deaf adolescents in Ghana. <https://doi.org/10.1002/hpm.2679> | Excluded | Wrong population | N/A |
| Shank & Foltz (2019) | Health and Wellbeing for Deaf Communities in Wales: Scoping for a Wales-Wide Survey. | Included | N/A | Katherine Rogers / March 2024 |
| Sheppard (2014) | Deaf adults and health care: Giving voice to their stories. | Included | N/A | Katherine Rogers / March 2024 |
| Sheppard & Badger (2010) | The lived experience of depression among culturally Deaf adults. | Included | N/A | Katherine Rogers / March 2024 |
| SignHealth (2014) | A Report into the health of Deaf people in the UK. | Included | N/A | Katherine Rogers / March 2024 |
| SignHealth (2022) | Review of the NHS Accessible Information Standard. <https://signhealth.org.uk/wp-content/uploads/2022/02/Review-of-the-NHS-Accessible-Information-Standard-FINAL.pdf> | Excluded | Wrong population | N/A |
| Sirch et al (2017) | Communication difficulties experienced by deaf male patients during their in-hospital stay: findings from a qualitative descriptive study. | Included | N/A | Katherine Rogers / March 2024 |
| Smeijers et al (2020) | Specialized outpatient clinic for deaf and hard-of-hearing patients in the Netherlands: Lessons learned in an attempt to improve health care. <https://doi.org/10.1111/jep.13369> | Excluded | Wrong population | N/A |
| Sousa et al (2020) | Barreiras de comunicaÃ§Ã£o com surdos no atendimento em saÃºde: um estudo descritivo. <https://doi.org/10.33233/eb.v19i1.2055> | Excluded | Non relevant language | N/A |
| Steinberg et al (2002) | Deaf women: experiences and perceptions of healthcare system access. | Included | N/A | Katherine Rogers / March 2024 |
| Steinberg et al (2006) | Health care system accessibility. Experiences and perceptions of deaf people. | Included | N/A | Katherine Rogers / March 2024 |
| Swannack (2018) | Deaf Futures: Challenges in Accessing Health Care Services | Included | N/A | Katherine Rogers / March 2024 |
| Tamaskar et al (2000) | Preventive attitudes and beliefs of deaf and hard-of-hearing individuals. | Included | N/A | Katherine Rogers / March 2024 |
| Tsimpida et al (2018) | Barriers to the use of Health Services among Deaf and HoH adults in Greece: A cross-sectional study. <http://dx.doi.org/10.5750/ejpch.v6i4.1566> | Excluded | Wrong population | N/A |
| Ubido et al (2002) | Inequalities in access to healthcare faced by women who are deaf. <https://doi.org/10.1046/j.1365-2524.2002.00365.x> | Excluded | Wrong study design | N/A |
| Witko et al (2017) | Deaf New Zealand Sign Language users' access to healthcare. | Included | N/A | Katherine Rogers / March 2024 |
| Witte & Kuzel (2000) | Elderly deaf patients' health care experiences. | Included | N/A | Katherine Rogers / March 2024 |
| Yabe et al (2020) | Healthcare providers' and deaf patients' interpreting preferences for critical care and non-critical care: Video remote interpreting. | Included | N/A | Katherine Rogers / March 2024 |
| Young et al (2016) | Understanding dementia: effective information access from the Deaf community's perspective. <https://doi.org/10.1111/hsc.12181> | Excluded | Wrong outcome | N/A |
| Zazove et al (1993) | The health status and health care utilization of deaf and hard-of-hearing persons. <https://doi.org/10.1001/archfami.2.7.745> | Excluded | Wrong population | N/A |
