# Supplementary material for: What are Deaf sign language users’ experiences as patients in healthcare services? A scoping review
Source: PLOS Glob Public Health. 2025 Feb 26;5(2):e0003535. doi: 10.1371/journal.pgph.0003535 (PMC11864532; doi:10.1371/journal.pgph.0003535)
Supplement: S2 Table — (DOCX) [file pgph.0003535.s002.docx]

S2 Table. Study characteristics

| **Study ID** | **Authors (year)** | **Publication type** | **Country** | **Aim of the study** | **Study design** | **No. of participants** | **Age** | **Gender** | **Inclusion / exclusion criteria** | **Intervention** | **Comparisons** | **Outcome data / results** | **CCAT Total score out of 40 / %** |
| --- | --- | --- | --- | --- | --- | --- | --- | --- | --- | --- | --- | --- | --- |
| **ID001** | Adigun et al (2020) | Journal article | Nigeria | Explore the experiences and satisfaction of pregnant deaf women with antenatal care in Nigeria. | Qualitative study: one-to-one interviews | 9 | 22-37 years old. Mean age = 29.5 yrs old | Female | (i) sign language user; (ii) aged 18-45 yrs; (iii) registered and attended antenatal clinics in the 6 months prior to the study period; (iv) willing to take part in the study | Antenatal care | N/A | Communication issues - found it difficult to interact with professionals. Health system related factors - non-inclusive health system contributed to deaf women's late attendance at antenatal clinics. (i) communication issues with health care workers (primarily done verbally); and (ii) attitudes of the health care professionals towards deaf women (e.g. stereotyping and labelling). | 30 out of 40 / 75% |
| **ID002** | Anderson et al (2017) | Journal article | USA | Explore Deaf trauma survivors' help-seeking experiences and elicit their recommendations for improving Deaf behavioural health services in Massachusetts. | Qualitative Interviews. Included Qs from the Life Events Checklist (Blake et al., 1995) and the PTSD Symptom Scale Interview. | 16 | 21-34 years old = 3; 35-44 years old = 2; 45+ years old = 11. | 13 female and 3 male | (i) age 21 years or older; (ii) Massachusetts residency; (iii) self-reported cultural identity as Deaf or Hard-of-Hearing; (iv) self-reported ASL fluency; and (v) one or more experiences of trauma at some point in the participant's lifetime. Exclusion: Only adults unable to provide informed consent and prisoners were excluded. | N/A | N/A | FORMAL HELP-SEEKING: (i) Individual-level barriers and facilitators - did not seek for formal help because they were unaware of treatment options or how to access them. Provider-level barriers and facilitators -provider's lack of shared communication with the participant, failure or refusal or provide ASL interpreters, and lack of awareness of Deaf culture and history. Negative experiences with providers who struggled to remain supportive or neutral in client encounters. Intervention-level barriers and facilitators— limited success with clinical interventions that lack a trauma focus; lack sufficient case management support; lack support education/provision of information; and lack structure. Community-level barriers and facilitators—because of small community dynamics, fearing that their providers, ASL interpreters, or group therapy members would violate confidentiality. Systems-level barriers and facilitators-difficulty requesting or getting ASL interpreters services. Limited availability of Def specialised services and long-wait lists for the services that do exist. | 28 out of 40 / 70% |
| **ID003** | Berman et al (2013) | Journal article | USA | To assess baseline breast cancer knowledge and practices among a sample of D/deaf women recruited into a randomized controlled trial of a breast cancer education program developed for this population. | Quantitative: Baseline survey. 51 items and six domains (including cancer experience; access to health information and services; etc.). | 209 | 40-49=63; 50-59=45; 60-75=57; 75+=28 | Female | (i) female, (ii) 40+ years old, (iii) self-identifying as D/deaf or hard of hearing , (iv) not a breast cancer survivor, (v) with no more than a high school diploma. | N/A | N/A | Health communication patterns, decision-making preferences, sources of health information: (i) 72.8% reported being satisfied with the communication they had with their doctor. (ii) Physicians were the most frequently cited source of health information (58.4%), with 60.5% reporting having asked a physician for information or suggestions about how to protect their health in the prior year. Re Breast Cancer screening practices: (i) 42.7% had not been screened in the prior two years and were therefore noncompliant with respect to screening guidelines. Of these women 62.7% reported seeing their doctor in the prior year, and 69.7% expressed satisfaction with communication they had with their doctor. Not receiving a referral from the doctor was the most frequently noted reason for non-receipt of a mammogram in the previous year. | 30 out of 40 / 75% |
| **ID004** | Berman et al (2017) | Journal article | USA | Understanding the breast cancer knowledge and experience of d/Deaf women. | Mixed methods: Qualitative interviews with 7 women; and surveys with 22 women. | 29 | Interview: 52-88; Survey: <50=1; 50-59=4; 60-75=7; 75+=9 | Female | Breast cancer survivors; ASL users. | N/A | N/A | Interviews: (i) navigating the health care system: communication issues. Survey: Negotiating the health care system: (i) health communication, (ii) decision making, and (iii) sources of information and assistance. 91% expressed satisfaction with the communication they had with their own doctor, however half of the women reported that they had difficulty understanding their diagnosis, treatment, or what would happen during recovery. Most frequently this was reported because of needing a sign language interpreter but not having one (36%), ... perceiving that the doctor did not explain well (27%). | 31 out of 40 / 78% |
| **ID005** | Cardoso et al (2006) | Journal article | Brazil | To characterize the perceptions of people with severe or profound deafness about the communication process with professionals in context of their health care. | Qualitative study: interviews | 11 | predominant age range was between 31 and 50 years old. | 7 male and 4 female | Aged over 18 and communicated by LIBRAS | N/A | N/A | Understanding - quality of communication / lack of deaf awareness. Issues with understanding the written language - sometimes results in deaf people being less instructed. Need for mediation - configured in family, friend and professional interpreters. Feelings - Fear over communication barriers when accessing health care without a companion. Afraid that they will take the wrong medication and/or the disease will continue. Fear about being discriminated against because they are deaf. | 22 out of 40 / 55% |
| **ID006** | Cerilli et al (2023) | Journal article | USA | The objective of this study was to explore older Deaf adults’ experiences with end-of-life care and planning, including barriers and facilitators, through interviews. | Qualitative study: interviews | 11 | Average: 62.5 years old (SD=9.8) | six male and five female | Self-identified as Deaf; ASL user; and at least 55 years old. | Educational videos about end-of-life care | N/A | Participants knowledge of advance care planning showed knowledge gaps with a pre-test score average of 4.6 correct out of 15 questions (SD=2.6). (1) Lack of accessible communication diminishes the participants' understanding of and trust in information sources: (i) Healthcare communication challenges; (ii) Medical jargon; (iii) Medical mistrust; (iv) trustworthiness of online resources. (2) ASL users' personal experiences and social networks facilitate an understanding of death and advance care planning: (i) Family and Media Exposure; (ii) Deaf-Centred Education. (3) Advance care planning education in ASL can improve understanding of end-of-life care for deaf ASL users: (i) Culturally Relevant Education; (iv) ASL Advance care planning information. | 23 out of 40 / 58% |
| **ID007** | Chin et al (2013) | Journal article | USA | To understand how ASL-using Deaf mothers learn about infant feeding and to identify their breastfeeding challenges. | Qualitative study: Community-based participatory research approach - did four focus groups. | 15 | Not reported | Female | (i) Culturally Deaf; (ii) used ASL; (iii) had a child 0-5 years. Method of infant-feeding was not a criteria for inclusion. | N/A | N/A | Despite the non-targeted recruitment materials, all 15 had initiated breastfeeding. Five cultural features of Deaf community interaction and language that supported breastfeeding success: (i) ASL as a Visual Language - e.g. such detailed explanations of problems in b/f are potentially more amenable to problem solving than spoken language descriptions. - e.g. signers "struggle" to describe how they learned to breastfeed. (ii) Deaf Cultural norms - placing a high value on the exchange of information. (iii) Use of technology - to both increase their information about breastfeeding and to reach out for support from other mothers. (iv) Language Access - to health providers - physicians, nurses, doulas, and lactation consultants - supported by certified sign language interpreters. Additionally - several practicing physicians are sign language fluent. (v) Self-Advocacy - majority of mothers are well educated - proactive in asking for help from health professionals. | 26 out of 40 / 65% |
| **ID008** | Costa et al (2018) | Journal article | Brazil | To identify the perception of the deaf woman regarding nursing care during pregnancy, childbirth and postpartum. | Qualitative: An exploratory-descriptive study with a qualitative approach - interview | 9 | Aged between 27 and 43 years | female | (i) Enrolled in the ADEMOC; (ii) residents of the urban area of Montes Claros/MG, (iii) historical Gestation, and (iv) LIBRAS user | N/A | N/A | Nursing team performance in the care process: Little contact with the nursing team during gestation. Barriers in communication with the nursing team | 24 out of 40 / 60% |
| **ID009** | Ferguson et al (2003) | Journal article | USA | To identify communication barriers and needs for Deaf and HOH patients when they seek pharmaceutical care, …. | Qualitative: Focus groups | 20 | Under 50 = 3; 51-60= 5; Over 60 = 12 | 11 female and 9 male | (i) Deaf/HOH patients older than 18 years, (ii) using at least two long-term prescription medications, (iii) ASL user. | N/A | N/A | Communication issues: did not understand the roles and responsibilities of the different types of people working in the pharmacy. Had to advocate for their communication needs, and voiced frustration over others' lack of understanding. Unable to hear their name being called, etc. Challenges of written communication. Medication use and safety concerns: more clarity is needed for medication instructions provided to Deaf people.…. Information resources: some acknowledged that their physicians were their primary sources of information about their medicines. Satisfaction with pharmacy visits and services: Unaware of the differences among all the staff working in the pharmacy. Meeting different staff members during different visits, making communication difficult. Unaware that many pharmacists can take blood pressures and provide services such as diabetes education. Most reported staff had attitude issues - appearing rushed and impatient. Some reported being generally satisfied with their visits to the pharmacy despite their experiences and struggles with communication - they said they were satisfied if they received the medication they needed. | 27 out of 40 / 68% |
| **ID010** | Fernandez-Valderas et al (2017) | Journal article | Spain | To explore the experiences of deafblind people in relation to health care throughout their lives. | Phenomenological qualitative study, through semi-structured interviews | 8 | mean age of 41.4 | 4 female and 4 male | Recruited from the APASCIDE - comprehensive care for Deafblind people. | N/A | N/A | (i) Accessibility and infrastructures - including insufficient lighting. (ii) Commented that it would be good if professionals had greater sign language skills. (iii) Human resources … (iv) Waiting time - believe it is longer when they do not previously know the professional or when there is no interpreter, etc. (v) Lack of privacy about intimate issues and prefer not to change health professionals. | 19 out of 40 / 48% |
| **ID011** | Foltz & Shank (2020) | Journal article | Wales, UK | Present reflections and opinions on health emergency services expressed by Deaf people in Wales - focus on texting 999 and interpreter availability. | Focus groups | 15 | 41-90+ | 11 female and 4 male | Not reported | N/A | N/A | Note: from the main study - Shank & Flotz (2019). Patients experience difficulty of communicating in English with 999 services, especially in stressful situations. | 15 out of 40 / 38% |
| **ID012** | Gichane et al (2017) | Journal article | South Africa | To assess pregnancy experience, utilization of maternity services and pregnancy outcomes amongst signing Deaf women in Cape Town, South Africa and compare findings to the general population of women int eh Western Cape and South Africa. | Mixed methods: Interviews which was recorded both in quantitative and qualitative items. | 42 | 18-49 years old | Female | (i) Adults of reproductive age (18-49); (ii) been pregnant at least once; (iii) native SASL users; and (iv) self-identify as Deaf. | N/A | N/A | (i) Antenatal care use and delivery: 93% received antenatal care when pregnant with youngest child. 59% had 4 or more antenatal visits during their most recent pregnancy. (ii) Poor communication in maternal services - while 2/3 responded that they had a good or okay experience, their explanations pointed to challenges. Communication was a major determinant of quality. 38% mentioned language barriers and lack of interpreting services. (iii) Staff behaviour at health facilities - 1/3 experienced kindness and helpfulness from staff - few discussed how providers were aware of their deafness and made accommodations. 15% disclosed very negative experiences with staff - inc rudeness from nurses, neglect, and yelling. (iv) Recommendations - 76% suggested that the health care service provided interpreters and/or that health care staff receive training in basic sign language. 16% - doctors/nurses provide better treatment and respect Deaf people. | 25 out of 40 / 63% |
| **ID013** | Gilchrist (2000) | Thesis | USA | To describe and interpret prevocationally deaf people's experiences communicating with nurses, the meaning of health, and their attitudes toward nurses. | Qualitative, hermeneutic phenomenological methodology was used. Interview via ASL interpreter. | 11 | 26-76 years old. | 5 female and 6 male. | Not reported. | N/A | N/A | Four metathemes: (i) Portraits of Deafness - description about participants; (ii) Seeing the words - facilitating interpreters; left in the dark; finding interpreters; wanting a specific interpreter; not wanting an interpreter; asking questions; (iii) seeing ourselves - Unfolding health; obtaining health care information; and wanting to know; and (iv) seeing nurses - explaining everything; nurses going out of their way; nursing activities; body language; rolling eyes; writing to communicate; fooling nurses; improving communication; presenting options. | 25 out of 40 / 63% |
| **ID014** | Hubbard et al (2018) | Journal article | USA | Conducted a pilot, descriptive study exploring women's prenatal, labor, and postpartum experiences. | Qualitative interview | 5 |  | female | (1) 18+ years old; (2) had a live birth in the past 6 months to 5 years, and (3) used ASL as their primary form of communication. Excluded - if had been hospitalised in the past 3 months. |  | N/A | Collaboration with ASL interpreters. - benefits of having consistent interpreters throughout prenatal care, inpatient labour and birth, and inpatient postpartum care. Missed opportunities for safe and individualised care using technology. - e.g. none took prenatal classes, absence of available technology in the hospital for deaf people (e.g. deaf alert for baby crying), use of VRS, etc. Potential quality improvement initiatives. - inc newborn hearing screen process - the manner it was carried out in and how results are communicated to parents is a significant patient satisfaction issue; and the wait time for interpreters. | 17 out of 40 / 43% |
| **ID015** | Iezzoni et al (2004) | Journal article | USA | To understand perceptions of health care experiences and suggestions for improving care among deaf or hard-of-hearing individuals. | Four group interviews - 2 in ASL for Deaf individuals, and 2 using Communication access real-time translation for HoH individuals. Men and Women were interviewed separately. | 14 deaf adults and 12 HoH adults | deaf - 23-51 years old; HoH - 30-74 years old | deaf - 8 female and 6 male. HoH - 6 female and 6 male. | Not reported. | N/A | N/A | 6 themes: (i) Conflicting assumptions about deafness - undermined patient-physician relationships. Meaning some physicians do not adequately respect patients' intelligence, motivation, and desire to understand and participate in their health care. It is thought that physicians do not comprehend the richness of Deaf cultural and linguistic identities. (ii) Conflicting perceptions of communication modalities - reported being expected to reading lips, writing notes, or bringing family members to interpret. (iii) Inadequate communication has consequences. - e.g. not understanding therapeutic regimens, etc. (iv) Communication during physical examinations and procedures - e.g. feeling discomfort and deaf people not being able to see clinicians during physical examinations, etc. (v) Interacting with office staff - e.g. not hearing name being called in waiting room. (vi) telephone communication. | 25 out of 40 / 63% |
| **ID016** | Jacob et al (2021) | Journal article | Malaysia | Explored the views on a proposed mobile health (mHealth) app in terms of design and features, that will be able to bridge the communication gap between community pharmacists and DHH patients. | Two focus groups in Malaysian sign language (BIM) | 10 DHH | 25-47 years old. | Of 10, only 1 was male and 2 declined to provide further demo info. | Deaf or HoH; aged 18 years and above. | mHealth app | N/A | (i) challenges and scepticism of the healthcare system - e.g. challenge of communicating with pharmacists and experiencing miscommunication, etc.; and (ii) features of the mHealth app. | 24 out of 40 / 60% |
| **ID017** | Jacobs et al (2021) | Journal article | USA | To establish a prevalence of hysterectomy among deaf women and provide insight into the experiences of those who have undergone hysterectomy. | Mixed method study - quantitative data from online Patient-Reported outcomes survey and reproductive health Qs from NHANES; and interviews with sample of deaf women. | 195 completed the survey - 67 underwent hysterectomy. 8 were interviewed. | Of 195 women: 35-49=27; 50-64=90; 65-74=55;75+=34 | Female | Not reported | N/A | N/A | If communicating with the doctor using written English or other methods increased the odds of hysterectomy (1.250 (0.521,3.001); p-value=0.62). Of those 8 who were interviewed, 5 reported good to excellent re communication with their OB/GYN and 3 reported poor to fair. Four used sign language interpreters - all underwent hysterectomy after 1990 when ADA was passed. Satisfaction of the quality and quantity of information received - 3 were fully satisfied, two felt little or not satisfied, and 3 felt somewhat satisfied. Those who had access to their preferred communication felt that they had being able to better understand hysterectomy and its process. Physician's approach to communication and demonstrating respect for patients is an important element in full communication access. Social support - inc having opportunity to listen to deaf friend who have already undergone the proceedure helped them feel confident. Access to information | 30 out of 40 / 75% |
| **ID018** | James et al (2022) | Journal article | USA | To describe emergency department (ED) communication experiences of Deaf patients who have used Eds in North Central Florida within the past 2 years. | Qualitative interview. Descriptive qualitative design - applied a transformative paradigm lens. | 11 - all have been interviewed. | Med=35 years (range 31-60) | 6 female and 5 male | (i) Self-identified as being DHH; (ii) ASL as their primary form of communication; (iii) 18 years or older; (iv) have been a patient, or have been the parent of a paediatric patient in the ED during the past 24 months. | N/A | N/A | Five themes: (i) requesting communication access can be stressful, frustrating, and time-consuming - inc (a) negotiating access; (b) requests fulfilled with VRI; and (c) friends and family as proxy interpreters; (ii) perspectives and experiences with Video Remote Interpreting - inc diminished patient-centred care and communication; and (in)appropriateness of VRI; (iii) expectations, benefits, and drawbacks of using on-site ASL interpreters - inc (a) increased patient engagement; (b) benefits of onsite interpreters over VRI; c) interpreter behaviour that harms patient-provider communication; (iv) written and oral communication provides insufficient information to Deaf patients - inc discharge instructions are inaccessible; and (v) ED staff and providers lack cultural sensitivity and awareness towards Deaf patients. Inc inconsistent policy application. | 30 out of 40 / 75% |
| **ID019** | James, Panko, et al (2023) | Journal article | USA | The aim of this descriptive study was to report the prevalence of communication accommodations used by DHH patients, segmented by patients' language preference. | Mixed methods study: Online survey and interview | 234 ASL users and 238 bilingual ASL/English users | ASL users - Mean age=34.3 (SD=5.5) and ASL/English users mean age = 35.3 (SD=5.5) | Female | At least 21 years old; given birth to at least one child within the 10 years preceding the survey; gave birth to most recent child in the USA; and have a hearing loss before the birth of most recent child. | N/A | N/A | Communication accommodations requested and provided during prenatal care; labour; and delivery. For prenatal - those who requested onsite interpreters; 5% did not get interpreters; and 6-7% received VRI despite it not being requested. For labour/delivery: onsite interpreters were requested higher than VRI for ASL users (64.1% vs 7.3%) and bilingual (56.3% vs 8%). Those who requested for onsite interpreters; 10% ASL users and 16.4% of bilingual participants did not get interpreters during the birth of their child. Experiences with VRI: connectivity issues were the most frequently report issues with VRI followed by the delay in setting up VRI and poor placement. | 25 out of 40 / 63% |
| **ID020** | James, Sullivan, et al (2023) | Journal article | USA | To qualitatively assess DHH patients' experiences with seeking emergency department (ED) care and how care was provided in the ED. | Qualitative interview | 4 DHH ASL users and 6 English speakers DHH | DHH ASL: 18-44=3; 45-64=1. English speaker DHH: 18-44=1; 45-64=2; 64+= 3 | DHH ASL: 1 male & 3 female; English speakers DHH: 1 male & 5 female | DHH: 18 years old or older; been patient in ED within 36 months before the interview. | N/A | DHH ASL / DHH English speakers | Patient-centred ED care differs between DHH ASL users and DHH English speakers: (1) Patients feeling stereotyped; (2) Being involved in the care process; (3) pain communication; (4) accommodations and patient-activated interpersonal and environmental modifications; (5) discharge processes and post discharge information needs. | 31 out of 40 / 78% |
| **ID021** | Kushalnagar et al (2019) | Journal article | USA | Investigates the trends of deaf patients' use of and satisfaction with the quality of VRI technology service in health settings. | Quantitative online survey. Paper focused on responses to 3 questions link to patients' opinions and experiences with VRI. | Only focus on participants who have actually used VRI in the past year - N=555. | Mean age = 45 years (SD=18 years) | Of avail info, 302 female and 243 male | (i) ASL as a primary language; (ii) age of 18 years or above; (iii) presence of bilateral hearing loss. | VRI | N/A | (i) 41% reported the quality of VRI as satisfactory. (ii) Those who have a regular health care provider are more likely to be dissatisfied with the quality of VRI service. (iii) Those who reported that VRI interfered with disclosure of health info to their health care provider were less likely to be satisfied with the quality of VRI service. ....... | 31 out of 40 / 78% |
| **ID022** | Kyle et al (2013) | Report | UK | To assess the current health of the Deaf BSL-using community in the UK, and to determine the link between their health status and the issues they face in communication and thereby their access to health care. | Mixed method study: Health check assessment. Inc: interview on use of GP services. | Health check assessment = 298. Interview = 47 BSL users | Health check assessment: 18-24=8; 25-44=113; 45-64=122; 65-82=55. Interview: | Health check assessment: 159 female and 139 male | BSL users. Interviewed those who took part in health check assessment. | N/A | N/A | Access to health services; (a) contact with primary care health services - Deaf people are more likely to express a preference to see a specific doctor than hearing people; 45% said they made appointments in person compared to 32% of GPPS. 44% reported difficult or very difficult in making contact with the GP. Receptionist - nearly 40% found them not very helpful or not at all helpful compared to 8% of GPPS. 65% were able to see GP within 2 days compared to 84% of GPPS. (ii) Experience of consultations: Doctors giving enough time - Deaf 66% compared to 88% GPPS rated as good or very good. Deaf likely to rate the explanations by the GP poorer than in GPPS. (iii) methods of communication: fewer deaf people reported confidence and trust (25%) compared to GPPS (67%). | 27 out of 40 / 68% |
| **ID023** | Lee et al (2021) | Journal article | Australia | To identify barriers and facilitators to access and communication for deaf individuals and Auslan interpreters in Australian general practice settings. | Qualitative Interviews. | 4 deaf participants | Deaf: 20-30=1; 30-40=2; 65+=1 | Deaf : 2 female and 2 male | Deaf participants: (i) hearing-impaired or profoundly deaf; (ii) used Auslan as their preferred mode of communication; (iii) culturally identified as a part of the Deaf community; (iv) older than 18 years of age. | N/A | N/A | From Deaf participants: (i) approachability/ability to perceive: e.g. English literacy as barrier to accessing and understanding health promotion material. (ii) Availability and accommodation: e.g. phone-based appointment systems as a barrier, and some reported that VRI was not always used to its full potential. (iii) Appropriateness/ability to engage: e.g. lack of cultural awareness of providers, and lack of provider knowledge about how to use interpreters. | 23 out of 40 / 58% |
| **ID024** | MacKinney et al (1995) | Journal article | USA | To examine the effectiveness of a special program for the deaf in improving ambulatory health outcomes. | Mixed method study: Case-cohort design. Interviews including the use of questionnaires. | 90 in Deaf Services Program (DSP) vs 85 Control from Deaf community | DSP: Mean = 47 years old (SD=18) | DSP: 44% male | DSP: adults receiving any care through the DSP. Control: not receiving any care at the DSP. | DSP | Control | Measures of health access and status were similar between DSP and Control, but DSP reported more office visits in the prior year and took more medication. (i) DSP more likely to use ADL for ambulatory physician visits - 84% used an ASL interpreter, while 6% use note writing - compared to 20% used ASL and 67% used note writing. Without an ASL interpreter, nearly 75% of both groups reported that their understanding of their providers was fair or poor. DSP reported more greater satisfaction with their communications with their physicians than did the controls. Increased satisfaction with physician communication was associated with increased compliance with Pap testing, mammography, rectal examinations, optometric examinations, and counselling in ASL, but not with breast examinations | 20 out of 40 / 50% |
| **ID025** | Miller et al (2019) | Journal article | USA | Examined whether deaf LGBTQ individuals patient centered communication and level of comfort in sharing health information in the presence of an interpreter contributed to coming out to providers. | Quantitative: Online health survey. | 313 | 18-75 years old. Mean=36.91 (SD=11.96) for Gay/Lesbian; Mean=31.38 (SD=10.68) for Bisexual/Queer | Gay/Lesbian: 101 male; 71 female; 12 genderqueer; Bisexual/Queer: 29 male; 84 female; 16 genderqueer | (i) ASL user. Exclusion criteria: (i) under the age of 18 years old; (ii) having unilateral hearing loss. | N/A | N/A | (i) Earlier coming out age in the transgender genderqueer-identified group compared to cisgender male/cisgender female. (ii) factors that were found to be significantly associated with the decision to disclose LGBTQ identities to healthcare providers were: (a) being accepted by others with whom one is close; (b) self-identification as a cisgender male: (c) self-identification as gay or lesbian; (d) high perceived patient-centred communication; (e) older age; (f) better health status; and (g) marital status. (iii) discussion of health information with interpreter presence is one of the predictors with LGBTQ disclosure-to-provider as an outcome. (iv) Patient centred communication but not sharing health issues with providers in presence of interpreters was associated with coming out to providers. - suggested that providers ability to deliver high-quality patient centred communication care had greater impact on the deaf patient's decision to disclose sexual orientation and/or gender identity. | 31 out of 40 / 78% |
| **ID026** | Mussallem et al (2022) | Journal article | USA | To describe the deaf population's experience with telehealth visits and identify key barriers and facilitators to its use. | Quantitative: Online survey. | 74 who did experience a telehealth appointment completed the survey. | 37.4 years old ±14.4 years. | 23% male | Not reported. | Telehealth appointments | N/A | (i) 66% experienced challenges with communication accessibility during the telehealth appointments. (ii) 1/3 reported needing to use their residual hearing to communicate with providers. 54% used an interpreter during the appointment. 1/3 used VRS that employs community interpreters who are not required to maintain general certification instead of a remote interpreter with specialised healthcare interpreting certification. 97% preferred to be able to see their healthcare provider on a video screen. - but 27% reported not being able to view their healthcare provider during the appointment. Participants with interpreters were more likely to say that they liked the privacy of telehealth visits. Participants with interpreters were more likely to report that they did not have quicker access to care but were less likely to report concerns about quality of health care received. 18% felt that the quality of health care received via telehealth is comparable to in-person visits; 38% felt that telehealth will never meet the quality of an in-person visit and 42% felt that telehealth is only good for the initial consultation and/or routine care. | 18 out of 40 / 45% |
| **ID027** | Myers et al (2022) | Journal article | USA | The objective of this paper is to address this gap for North Carolina through a mixed-methods study exploring quantitative data on preferred and actual modes of communication and their outcomes, together with qualitative data exploring further nuances of communication accessibility. | Mixed methods study: Online survey and interview | Online survey = 189; interview = 54 | 18-34=24; 35-64=129; 65+ =36 | 46 male and 143 female | Not clear reported but all participants were ASL users and who received services, newsletters, or worked for the North Carolina Department of Health and Human Services Division of Services for DHH. | N/A | N/A | While 45% of respondents used a professional sign language interpreter, 65% of respondents preferred to do so. Accessible communication was associated with lower odds of dissatisfaction with communication (OR = .19, p < .05). Dissatisfaction with communication was associated with greater odds of unmet need for healthcare (OR = 8.95, p < .05). Interview respondents emphasized their preference for on-site interpreters, explaining how video remote interpreting was subject to technical difficulties while writing back-and-forth led to missing important information. | 23 out of 40 / 58% |
| **ID028** | Napier et al (2013) | Journal article | Australia | To gain an in-depth understanding of whether deaf Australians who use Auslan as their primary language feel that they can sufficiently access preventative and on-going healthcare information, and how essential they feel it is to access information in Auslan. | Qualitative: A phenomenological, inductive study, with data collected via an interviews. | 72 | 18-35=15; 36-50=18; 51-65=14; 66+=19. unknown=6 | 62.5% female | Deaf Auslan users. | N/A | N/A | Four major themes: (i) level of English literacy; (ii) inadequate access to health care interpreting services; (iii) inaccessible health-related services; and (iv) limited access to other sources of information. But this paper focuses on the first theme: level of English literacy. 9/72 expressed confidence in their English literacy skills. 11 participants said they could follow what they read but preferring text that was presented in bullet points or had accompanying images. All participants feelt more comfortable when information was accessible in Auslan. DeafBlind also face additional challenge of web-based information not being as easily accessed. | 22 out of 40 / 55% |
| **ID029** | Napier et al (2014) | Book Chapter | Australia | To focus on the Australian context and examine the nature of Deaf Auslan users' access to preventive and ongoing healthcare information in particular. | Qualitative: Interviews | 72 | 18-35=15; 36-50=18; 51-65=14; 66+=19. unknown=6 | 45 (62.5%) female and 27 (37.5% were male. | Deaf Auslan users. | N/A | N/A | Themes include those that provide their insight into access to preventive and ongoing healthcare information: (i) English literacy; (ii) Attitudes toward preventive or ongoing health care; (iii) Access to interpreters for preventive and ongoing healthcare services; (iv) Access to interpreters for primary health care; (v) Communication preferences; (vi) Access to general healthcare information; (vii) Sources of general healthcare information. | 27 out of 40 / 68% |
| **ID030** | O'Hearn et al (2006) | Journal article | USA | Compares deaf and hearing women's experiences in prenatal care, in particular as related to communication and patient satisfaction. | Quantitative: Survey questionnaire - adapted from Omar and Schiffman's prenatal satisfaction measure. | 23 deaf women and 32 hearing women who had a baby within the past 3 years completed the questionnaire. | Deaf: >25=0; 25-29=22; 30-34=39; 35-39=35; 40+=0; not answered=4. | Female | Not reported other than all participants had a baby within the past 3 years. | N/A | N/A | Hearing women have more prenatal appointments than deaf women. Hearing women getting significantly more information from their doctors than deaf women. Overall satisfaction with prenatal care - hearing women had higher satisfaction scores than deaf women. Hearing also reported greater satisfaction with communication and with perceived physician concern than deaf women. Deaf women became less satisfied overall as the number of prenatal appointments increased - the same was not found for hearing women. 95% deaf preferred their doctor to communicate in sign language or via an interpreter, but only half reported being provided with a professional interpreter at least some of the time. Interpreter expectations were met and exceeded; satisfaction increased. Importance of clear communication was the subject of many deaf respondents' comments. | 18 out of 40 / 45% |
| **ID031** | Oliveira et al (2015) | Journal article | Brazil | describing the accessibility of the deaf to public health services | Interview - qualitative | 11 | Age range: 23-44 | 7 male and 4 female | deaf people above 18 years old; who communicate by sign language; with cognitive conditions that allowed interaction with one of the bilingual researchers | N/A | N/A | (i) Geographical and Organisational accessibility: (a) Strategies used in accessibility for deaf people; (b) obstacles perceived; c) Professional interpreter of Libras at health services; (ii) Socio-Cultural accessibility - feeling of exclusion/discrimination. | 18 out of 40 / 45% |
| **ID032** | Panko et al (2022) | Journal article | USA | Objective: Women who are deaf experience higher rates of reproductive healthcare barriers and adverse birth outcomes compared with their peers who can hear. This study explores the pregnancy experiences of women who are deaf to better understand their barriers to and facilitators of optimal pregnancy-related health care. | Qualitative study: interviews | 45 deaf ASL users | mean age = 35. Age range: 22-44 years old | All female | Female aged 21-50 years old and have given birth within the past 5 years in the USA. | N/A | N/A | Three major themes emerged: (1) communication accessibility; (2) communication satisfaction; and (3) healthcare provider and team support. Common barriers included choosing healthcare providers, inconsistent communication access and difficulty accessing health information. However, when women who are deaf were able to use ASL interpreters, they had more positive pregnancy and birth experiences. Self-advocacy served as a common facilitator for more positive pregnancy and healthcare experiences. | 23 out of 40 / 58% |
| **ID033** | Parise (1999) | Thesis | Canada | To explore two complementary aspects regarding deaf people and access to health care. The first facet of the study explored the health care experiences of deaf people. The second aspect of the investigation identified the strategies and resources used by deaf persons to cope with the present gaps in the health care system and constraints encountered. | Individual interviews and focus group interviews. | 10 | 30-39=3; 40-49=5; 50-59=1;60-69=1 | 3 female and 7 male | (i) use sign language; (ii) at least 19 years of age; (iii) be able to provide detailed experiential information about their experiences with the health care system; (iv) agree to a videotaped interview. | N/A | N/A | Three main themes: (i) the health care system as a source of alienation and suffering; (ii) communication as the link to both worlds; and (iii) marginalisation as a stimulus to look within. | 22 out of 40 / 55% |
| **ID034** | Pereira & Fortes (2010) | Journal article | Brazil | To describe and analyse deaf (LIBRAS-using) patients' perception of the process of interpersonal communication and information in both private and public health care assistance in Sao Paulo, Brazil. | Qualitative interview | 25 | not reported | not reported | (i) deaf; (ii) users of LIBRAS; (iii) users of the private or pubic health system; (iv) age 18 years or older; (v) willing to take part. | N/A | N/A | Commonly expressed criticisms: (i) communication difficulties (or even an absence of communication) between patients and health services staff; (ii) culture clash and a harmful inability among the service providers to distinguish among the roles of companions, caretakers, and professional translator/interpreters. Communication difficulties: a) stressful process of negotiation over the communication strategies that could best promote effective communication. b) realisation that a medical consultation is necessary means that a deaf person will have to have patience and make a large amount of effort. c) most of the time the deaf person had a negative representation of health professionals. d) serious problems in communication thus leaving the doctor's office with doubts, having been unable to express their feelings, symptoms, and the history of their medical needs. Role confusion about companions, caretakers, and professional interpreters - use of hearing relative (e.g.) barely share the information exchanged with the doctor and discouraging his or her autonomy. Cultural clash - misunderstanding and nonidentification of the patient as a member of the Deaf community. Knowledge gaps affect communication. Deprivation of information about health - demanded clearer and more accessible information. | 21 out of 40 / 53% |
| **ID035** | Pertz et al (2018) | Journal article | USA | Purpose was to design and pilot an accessible, integrated mental health program for the Deaf population, scalable for other health centers interested in serving these individuals. Inc - level of patient acceptance for utilizing telemental health-based appointments. | Mixed methods study - inc Individual and clinic level data were collected and analysed. | 50 | 18-61+. Mean age = 46.4 years | 26 female and 24 male | (i) Deaf; (ii) ages 18 years and older; (iii) used ASL; (iv) had family medicine and clinical social work services available to them. | Use of telemental health versus in-person visits was left to the patient's decision. | N/A | With regards to feedback on the use of telemental health services, they praised the option of having an opportunity to receive telemental health care remotely. Two participants who had lower satisfaction scores struggled with video quality issues. Frequently praised the option of having the opportunity to receive telemental health care remotely, as well as having health care provided by concordant language and cultural professionals. (i) language concordance; (ii) integration of health care providers; (iii) videophone access: | 23 out of 40 / 58% |
| **ID036** | Pinilla et al (2019) | Journal article | Germany | To explore disease concepts embedded in signs, primary non-communicable disease prevention behaviour and communication barriers among members of a deaf community. | Qualitative study - interviews. | 15 - 2 with history of diabetes and 13 without. | 20-65. | 5 female and 10 male | (i) use of sign language; (ii) deafness before the age of two; (iii) willingness to take part. | N/A | N/A | Four main themes: (i) General diabetes perception: (a) signs for diabetes differ according to the underlying concept of diabetes; (b) diabetes knowledge depends on personal experience and social environment; (iii) diabetes is perceived as a private and personal issue; (ii) Health information seeking behaviour depends on personal health status: (a) learning from a friend or having a disease yourself influences your knowledge; (b) the main source for health information is the internet and different online presentation modalities are used; (iii) Learning about general prevention: (a) parents and peers as most important hidden health promoters; (b) acute change in the personal health condition is a tigger to adopt a healthier life style; (iv) persisting communication barriers with health professionals: (a) sign language is the preferred way of communicating and deaf culture should be taken into consideration; (b) Get a sign language interpreter; (c) use supportive communication strategies. | 22 out of 40 / 55% |
| **ID037** | Reeves et al (2005) | Journal article | UK | To assess Deaf people's access to primary care. This paper concentrate on the issue of communication between Deaf people and general practitioners (GPs, family practitioners, primary care physicians) within the primary healthcare consultation process. | Mixed method study: data collected using two interviews (quantitative-qualitative): (i) main interview - inc Qs about experiences of using primary care services, and Qs about recent consolation with their GP; and (ii) A&E interviewee - those who attended A&E in recent past. | 98 | 18-34=26%; 35-49=27%; 50-64=29%; 65+=16% | 51% female and 49% male | (i) adults (18 years or older). (ii) Deaf sign language users. | N/A | N/A | (i) Preferences for communication support at consultations: 63% said it was very important for them to have someone present at consultations to help them communicate with the doctor. 54% expressed a preference for using a professional sign language interpreter, while another 31% preferred to use a family member. (ii) General perceptions about GPs: compared to NSP study sample, Deaf people less likely to have positive views about their usual GP. Deaf people were significantly less likely to agree that their GP listens to them, treats them with courtesy and respect, and does not make them feel they are wasting his/her time. (iii) Most recent GP consultation: a) companions at the consultation - majority were there at their last GP consultation. Only 17/98 had an interpreter. Those 17 people arranged an interpreter themselves. b) Quality of communication - those without communication support were asked how much of GP's communication had been clear to them - 52% felt most had been clear, 32% thought some was clear, 16% understood very little. 34% were not able to ask all their Qs; 35% were not certain about the doctor's management; 31% did not fully understand the advice on aftercare; and 37% gained no better understanding of their illness. (iv) Making complaints: 19% reported that they have complaint, and 21% said they wanted to complaint but did not. Compared to 1% of the NSP sample that have had a complaint, 11% wanted to but did not. 26% made at least one comment expressing concern about a prescribed medication although they were not asked about it. | 23 out of 40 / 58% |
| **ID038** | Rodriguez-Martin et al (2018) | Journal article | Spain | Aims: (i) to know the communication access difficulties of deaf people in healthcare context; and (ii) determine how these difficulties violate their rights. | Mixed method study: Qualitative - ethnography, field work with participant observation and non-participant observation, and in-depth interviews. | 25 | 18-75 | 18 female and 7 male | (i) deaf sign language user; (ii) aged 18 or over; (iii) willing to take part. | N/A | N/A | Two key themes: (i) barriers to information access - the right to information - a) adaptation of information; and (ii) communication barriers - the right to privacy and the right to decide. Communication with health professionals - difficulty of lip reading and writing notes. Use of interpreters. Consequences of bad communication - leads to uneasiness, discomfort and stress. And impact on health and violation of rights. | 25 out of 40 / 63% |
| **ID039** | Schniedewind et al (2020) | Journal article | USA | Describe the types, frequency, severity, and trends in healthcare access complications experienced by Deaf patients. | Quantitative: Retrospective review of complaints about interpreter provision in healthcare settings. | Not clear | 18-65+ | 64.8% female | Not clear. | N/A | N/A | 49.5% were promised an interpreter at their initial request. Reason for complaint varied: 48.2% were told that an interpreter was not available; 28.7% of interpreters provided were not sufficiently qualified to interpret; 18.5% were told that an interpreter would be secured but were not; and 4.6% were told that the provider was not taking new patients. 78.7% complaints were resolved. 83.3% involved medical clinics and 16.7% involved dental clinics. Significant relationship between complaints made from urban areas and being more likely to be promised an interpreter on initial request and less likely to be told that an interpreter was not available. Unqualified was marginally positively associated with complaints about medical clinics. Complaints from rural areas 82% less likely to have had their complaint resolved after mediation attempts by CDHH. | 29 out of 40 / 73% |
| **ID040** | Shank & Foltz (2019) | Report | Wales, UK | Pilot study to explore (1) the barriers and enablers to staying healthy in d/Deaf communities; (2) potential actions for different professional groups). | Three focus groups with Deaf people. | 13 | Not reported. | Not reported. | Not reported but all are BSL users living in Wales. | N/A | N/A | (i) Health and health behaviours: misdiagnoses and wrong treatments may easily occur due to miscommunication, especially in the absence of an interpreter. (ii) Barriers and enablers: few participants knew about the resources available; most participants reported getting their health info informally through their social network; and helping each other in the community when it comes to health information. (iii) Accessing health services: highlighted challenges in booking an appointment, and an interpreter. (iv) Hospitals and Emergency Services: reported difficulties in interacting with emergency services and hospitals. (v) Mental health: e.g. Access to mental health emergency but no interpreter. (vi) Doctor-Patient Relationship: all echo the need for Deaf awareness training among medical professionals. (vii) Use of technologies: reported to be open towards online services but said that their doctors were not using these services or were not aware of these services. | 13 out of 40 / 33% |
| **ID041** | Sheppard (2014) | Journal article | USA | To give voice to 9 Deaf adults as they describe their experiences accessing and receiving health care. Aims are to describe healthcare experiences of childhood, healthcare access barriers, and communication barriers. | Qualitative: The stories stem from interviews obtained during a qualitative study that explored depressive symptoms among culturally Deaf adults, in the words and language used by the culture. (Shepard and Badger, 2010, study) | 9 | 21-62 | 7 female and 2 male | (i) culturally Deaf and over the age of 18. | N/A | N/A | (i) Reflections of childhood health care - all recalled repeated visits to doctors as parents and healthcare providers sought reasons for deafness. (ii) Deaf adults as patients - all reported difficulty accessing health care and described their healthcare encounters as, often meaningless. Most expressed frustration with the healthcare system, and the lack of healthcare resources available to Deaf patients. Universal concerns included (a) not understanding how to obtain a referral for a specialist, and (b) being unable to locate a primary care or mental health provider willing to accept a Deaf patient. Many use a telephone relay system to contact an ambulance or try to get an appointment, which was frustrating. Most reported nurses, doctors, and office staff as being impatient. Most left the provider’s office with minimal understanding of the diagnosis and many were confused about treatment or follow up needed. Medication - rarely understood the drug's use or side effects. Often the focus of the visit became the etiology of deafness rather than the concerns of patient. None were asked about thoughts of suicide. Communication barriers - differences in body language and eye contact between providers and Deaf patients may contribute to misunderstandings and discomfort. Most concern about the confidentiality of an interpreter as some of them are friends to participants. | 27 out of 40 / 68% |
| **ID042** | Sheppard & Badger (2010) | Journal article | USA | To describe depressive symptoms experienced by Deaf adults, describe the ASL signs and phrases that express the Deaf adult's perspective of depression, and describe commonalities within the experience of depression among Deaf adults. | Qualitative interview: Exploratory study used a hermeneutic phenomenological methodology. | 9 | 21-65 years | 7 female and 2 male | Culturally Deaf and over the age of 18. | N/A | N/A | Four themes: (i) early emotional chaos; (ii) feeling depressed; (iii) reaching out; and (iv) Deaf-belonging. Reaching out: all shared a desire for help but were unsure how to reach out. Health care was described as embarrassing and frustrating. All prefer to have an interpreter present for most health care, 8 reported that an interpreter was unwelcome when seeking mental health - worried about the stigma. | 25 out of 40 / 63% |
| **ID043** | SignHealth (2014) | Report | UK | To find out about Deaf people's experience of healthcare, whether Deaf people have poorer health than the rest of the population, to suggest reasons for any inequalities, and to identify practical measures that could reduce them. Aim of interview - understand more about the Deaf person' experience of their health and health services. | Mixed method study: Three stages: (i) Online survey; (ii) Individual health assessments; and (iii) semi-structured interviews. Aim of interview was to understand more about the Deaf person's experience of their health and health services. | (i) 533 completed survey; (ii) 300 took part in individual health assessments; (iii) 47 were interviewed | Not reported | not reported | Not reported but Deaf and BSL users | N/A | N/A | Experience: access to health services: (i) The patient journey - a) making an appointment - 45% go to their GP in person to book an appointment; b) not making an appointment - 90% hearing people had not visited GP recently and said that this was because they had not needed to, compared to 30% of Deaf respondents. 36% didn't think it worth seeing their GP because the communication was poor. c) The consultation - Deaf people - 18.7% reported very good; clinician giving them enough time compared to 49% of general population. d) communication - big difference between how Deaf people wanted to communicate and what happened in practice. E.g. most wanted to use BSL (about 80%) but few did (about 30%). e) Satisfaction and trust in the doctor - e.g. 24.6% deaf people said yes to trust and confidence in their doctor compared to 67% of general population. f) Use of interpreters - reported of unqualified interpreters being used. (ii) Access to information: poor e.g. relying on written information. | 16 out of 40 / 40% |
| **ID044** | Sirch et al (2017) | Journal article | Italy | To explore the communication experience of deaf patients with regards to their in-hospital stay. | Focus group in Italian sign language | 9 | 30-60 years | Male | (i) deaf prior to language acquisition; (ii) older than 18 years of age; (iii) with no history of neurological and/or psychiatric comorbidity; and (iv) with at least one in-hospital experience over the last year. | N/A | N/A | Four main themes: (1) Experiencing a common vulnerability: the need of reciprocal understanding and sensitivity - ; (2) Being outside their comfort zone: feeling discriminated against once again; (3) perceiving a lack of consonance between care and needs - they reported difficulties in expressing and being recognised in their care needs; (4) developing a sense of progressive disempowerment - communication is the prevalent obstacle reported by deaf participants, threatening their discussion making participation and accessibility to healthcare difficult. | 31 out of 40 / 78% |
| **ID045** | Steinberg et al (2002) | Journal article | USA | Investigated the knowledge, attitudes, and healthcare experiences of Deaf women. | Qualitative: focus groups | 45 | 20 who did demographic info: age range+18-67 - median age of 42. | Female | (i) Female; (ii) communication preference of ASL, (iii) willingness to participate. | N/A | N/A | (i) Health knowledge and access to information - although 31% had completed their degrees and functioned as leaders within the Deaf community, most did not know the meaning or importance of preventative care, cancer screening, mammography, etc. Did not ask Qs to their physician because of uncertainty about what to ask. Lack of information cause distress and heightened discomfort. (ii) Negative imagery in medical vocabulary - (iii) Access to care and communication in the healthcare encounter - (a) Lack of common language between provider and Deaf women. (b) Healthcare providers with communication abilities and use of interpreters - some reported their willingness to drive long distances or be flexible with time schedules to have an appointment with practitioners who would take the time and effort to communicate with them. | 29 out of 40 / 73% |
| **ID046** | Steinberg et al (2006) | Journal article | USA | Explore experiences with health care in the Deaf community. | Qualitative: Focus groups | 91 | 24-83. Median age = 51 | 46 female and 45 male | (i) deafness; (ii) communication preference for ASL; (iii) willingness to share health care experiences. | N/A | N/A | (i) Quality of Health Care Communication - reported having a poor understanding of their clinicians' instructions because of difficulty with health care communication. Many preferred qualified interpreters but were infrequently available. Alternatives to an interpreter were often inadequate - e.g. speechreading, written communication, telephone communication, etc. (ii) Practitioners with Sign Language Skills - some from Rochester reported experiences communicating directly with clinicians in ASL - reports of these encounters were positive. (iii) Emotions Elicited by Health Care Experiences - many expressed strong emotions about their health care experiences - e.g. fear about consequences of miscommunication, and feelings of mistrust. Felt that some health care workers disliked working with deaf people. (iv) Knowledge of the Americans with Disabilities Act. (v) Suggestions for improvement - for practitioners to better understanding of deaf people and sign language, and how to work with an interpreter. (vi) Other positive experiences - Inc use of ASL by practitioners; when practitioners took time to explain and use visual aids, and when interpreter services were provided. | 25 out of 40 / 63% |
| **ID047** | Swannack (2018) | Master thesis | South Africa | To explore the narratives of Deaf individuals and accessibility to health care services. This study aims to better illuminate how Deaf people perceive the hearing-dominated health care system and how this affects their overall health and well-being. | Semi-structured interviews, photo-elicitation interviews, participant observation. | 6 | Age range: 25-56 | 3 female and 3 male | (i) Deaf South Africa; (ii) aged 25+ | N/A | N/A | (i) Stigma - associated with being Deaf. Participants expressed their frustration for not being recognised about their abilities, culture, and linguistic rights. (ii) Health care as a concern: concern about receiving adequate health care - inc not enough programs offered by deaf orgs about health care as well as no specialist training for interpreters working in health care. (iii) Perspectives on using SASLi for Communication - inc little availability and use of unqualified interpreters. Deaf patients with little or no knowledge of health care and low literacy can be an issue. (iv) relationship with health care professionals - unable to establish trusting relationship which can cause tension, stress and misinformation. The three most significant challenges encountered between the participants and practitioners are: 1) conflicting perspectives on being Deaf; 2) differing perspectives on effective communication strategy; and 3) risks presented by miscommunication. | 23 out of 40 / 58% |
| **ID048** | Tamaskar et al (2000) | Journal article | USA | To investigate the unique health care issues of deaf and hard-of-hearing persons by studying their attitudes, beliefs, and behaviours toward preventive medicine. | Quantitative: Survey. | 140 DHH and 76 hearing participants. | DHH: Mean age=51.1; Hearing: mean age: 42.3 | DHH: 45.3% male and hearing: 31.5% male | Aged 18 years or older. | N/A | N/A | DHH: 19.2% speak only, 20.8% speak more than sign; 33.1% sign more than speak; 26.9% sign only. (i) Use of health care services and health problems: DHH more likely to say they avoided physicians because of communication problems, interpreter unavailability, and other unspecified causes. (ii) Attitudes toward health maintenance behaviours - hearing more likely get information from their physician. Within DHH: Those who preferred ASL more likely than those who preferred spoken English to believe that regular screening for cholesterol level, blood pressure, and weight and height was important and less likely to believe that screening for hearing was important. (iii) Health maintenance procedures performed by physicians - DHH more likely to have vision examined and for women to have a mammogram. (iv) Beliefs about health behaviours - differences between hearing and DHH - e.g. hearing more likely to believe regular physical examinations, regular exercise, smoking less and watching weight improve overall health. Deaf more likely believe having 2 alcoholic drinks daily improve overall health. | 20 out of 40 / 50% |
| **ID049** | Witko et al (2017) | Journal article | New Zealand | To investigate deaf New Zealand Sign Language users' quality of access to health services. | Qualitative data - focus group, interviews, and online survey. Deaf people: N=32 took part in focus groups; n=9 individual interviews; 15 did the online survey. | 56 deaf participants | not reported | not reported | Not reported other than Deaf participants are NZSL users. | N/A | N/A | (i) Sensory barriers - making or change an appointment - only way is going in person as phone is not accessible. Even negotiating these encounters through speech and/or writing is difficult. Another barrier was communicating via an intercom. (ii) Use of interpreters - many reported that interpreters were not provided, leaving them feeling disempowered. Some reported that staff expected them to bring family members to assist with communicate with them. (iii) Consent and treatment compliance - felt that staff made no attempt to explain procedures that were about to happen or to ascertain consent. Some reported discontinuing treatment when they did not understand why they needed to take medication long-term or undergo follow-up procedures. (iv) Access to printed health information - many struggled to understand printed health information. (v) Cultural recognition - when deaf people were recognised by staff as being culturally deaf sign language users, they described feeling more satisfied with a health consultation even without an interpreter. Common annoyance was a sign reading 'hearing impaired' placed above their bed. Another example - newborn hearing screening and being told that her baby had passed the test. | 18 out of 40 / 45% |
| **ID050** | Witte & Kuzel (2000) | Journal article | USA | Seek to understand the health care experiences of elderly Deaf adults in Richmond, VA. | Focus groups. | Not reported. | Not reported. | Not reported | (i) aged 55+; (ii) Onset of hearing loss before age 10 years; (iii) involvement with Deaf culture; (iv) use sign language to communicate. | N/A | N/A | Reported difficulty communicating with the physician's office by the telephone. Reported using a TTY to communicate by telephone. But some reported office contacted them without using TTY or relay service. Having to rely on hearing family or friend to communicate by telephone. Communication in office - many reported frustrating experiences in the waiting room. Reported different ways: lip-reading with difficulty, writing things down, and use of interpreter (friend/family or professional). Most believed that hospitals or physicians wont hire an interpreter for them because of the costs, etc. Frustration for having to postpone tests or having to plan interpreters nearly a week before, etc. report of unskilled interpreters. | 14 out of 40 / 35% |
| **ID051** | Yabe et al (2020) | Journal article | USA | To identify healthcare providers' and DHH patients' interpreting preferences for VRI and in-person interpreting during critical care (e.g. surgery, urgent care) and non-critical care (e.g. follow up, non-urgent care). | Mixed methods study - online survey and qualitative interviews. 41 DHH patients completed online survey. Interviews - 8 DHH to explore online survey findings. | DHH: 41 did the survey; and 8 were interviewed. | DHH: 20-29=2; 30-39=5; 40-49=6; 50-59=9; 60+=8 | DHH: 22 female and 17 male did the survey; and 4 males and 4 females were interviewed. | DHH patients: (i) used VRI in clinical settings in the past 10 years; were 18 years or older; (iii) used ASL. | N/A | N/A | Five themes were identified, including positive and negative experiences for each theme: (i) Experiences; (ii) Preferences; (iii) Opinions; (iv) Suggestions; (v) Other concerns. DHH patients preferred in-person interpreting for critical care to obtain effective communication, translation accuracy, and better treatment. | 25 out of 40 / 63% |
