# Supplementary material for: What are Deaf sign language users’ experiences as patients in healthcare services? A scoping review
Source: PLOS Glob Public Health. 2025 Feb 26;5(2):e0003535. doi: 10.1371/journal.pgph.0003535 (PMC11864532; doi:10.1371/journal.pgph.0003535)
Supplement: Attachment — Submitted filename: Response to Editor.docx [file pgph.0003535.s006.docx]

**PGPH-D-24-01584R1**

**PLOS Global Public Health: What are Deaf sign language users’ experiences as patients in healthcare services? A scoping review**

**Response to PLOS Global Public Health**

| **Academic Editor Shaira Joy Petancio** | **My responses** | **Location of changes made** |
| --- | --- | --- |
| We've checked your submission and before we can proceed, we need you to address the following issues: As required by our policy on Data Availability, please ensure your manuscript or supplementary information includes the following: |  |  |
| 1. A numbered table of all studies identified in the literature search, including those that were excluded from the analyses.  a. For every excluded study, the table should list the reason(s) for exclusion.  b. If any of the included studies are unpublished, include a link (URL) to the primary source or detailed information about how the content can be accessed. | We have now produced new supplementary table (S1 Table – please note that the old S1 Table is now S2 Table), and included the information with authors, years, title of the papers, whether they were included or excluded; if excluded then reason for exclusion is stated for those 91 articles that have been assessed for eligibility.  URL links have been added for all those excluded studies in the ‘TITLE’ column. | Produced new table – S1 Table.  Please note the old S1 Table (on study characteristics) is now labelled as S2 Table. |
| 2. A table of all data extracted from the primary research sources for the systematic review and/or meta-analysis. The table must include the following information for each study:  a. Name of data extractors and date of data extraction  b. Confirmation that the study was eligible to be included in the review.  c. All data extracted from each study for the reported systematic review and/or meta-analysis that would be needed to replicate your analyses.  If data or supporting information were obtained from another source (e.g. correspondence with the author of the original research article), please provide the source of data and dates on which the data/information were obtained by your research group. | We have included the column with information of name of data extractors and date of data extraction in the S1 Table on full-text articles assessed for eligibility.  In the manuscript we have made it clear that the study characteristics table (S2 Table) only refer to studies that were eligible for inclusion and were in the review.  I can confirm that the data in the tables is sufficient for the review to be replicated.  We have added in the manuscript that 'no data or supporting information were obtained from another source other than available in the published sources.' | S1 Table.  Pg. 9  Pg. 7 |
| 3. If applicable for your analysis, a table showing the completed risk of bias and quality/certainty assessments for each study or outcome. Please ensure this is provided for each domain or parameter assessed. For example, if you used the Cochrane risk-of-bias tool for randomized trials, provide answers to each of the signalling questions for each study. If you used GRADE to assess certainty of evidence, provide judgements about each of the quality of evidence factor. This should be provided for each outcome. | There is a section on critical appraisal for all of 51 included items on page 10-12. We have now added new column in the S2 Table on study characteristics with information on CCAT score out of 40 as well as %. | S2 Table. |
| 4. An explanation of how missing data were handled. | There were no missing data. We have added the no missing data statement in the manuscript. | Pg. 7 |
